# Supplementary material for: Nutritional and Supplemental Interventions for Prevention and Treatment of Oral Mucositis in Pediatric Oncology
Source: Nutrients. 2025 Nov 11;17(22):3521. doi: 10.3390/nu17223521 (PMC12655015; doi:10.3390/nu17223521)
Supplement: Supplementary file 1 [file nutrients-17-03521-s001.zip › nutrients-3923468-supplementary File S1.pdf]

Scopus

TITLE-ABS-KEY (child* OR adolescen* OR pediatric* OR paediatric* OR "young patient*" OR teen*)

AND TITLE-ABS-KEY (cancer OR oncolog* OR malignan* OR neoplas* OR leukemia OR lymphoma OR "stem cell transplant*" OR "bone marrow transplant*")

AND TITLE-ABS-KEY ("oral mucositis" OR mucositis OR stomatitis OR "oral complication*" OR "mouth sore*")

== 4,715
time restriction
== 3,784
language:
== 3581
article type only
==3055

Pubmed

(child*[tiab] OR adolescent*[tiab] OR pediatric*[tiab] OR paediatric*[tiab] OR "young patient*"[tiab] OR teen*[tiab]) AND (cancer[tiab] OR oncolog*[tiab] OR malignant*[tiab] OR neoplas*[tiab] OR leukemia[tiab] OR lymphoma[tiab] OR "stem cell transplant*"[tiab] OR "bone marrow transplant*"[tiab]) AND ("oral mucositis"[MeSH Terms] OR mucositis[tiab] OR stomatitis[tiab] OR "oral complication*"[tiab] OR "mouth sore*"[tiab])

==1046
time restrictions
==860

Language
== 817

Study type

==218

Google Scholar:

TITLE-ABS-KEY (child* OR adolescen* OR pediatric* OR paediatric* OR "young patient*" OR teen*) AND TITLE-ABS-KEY (cancer OR oncolog* OR malignan* OR neoplas* OR leukemia OR lymphoma OR "stem cell transplant*" OR "bone marrow transplant*") AND TITLE-ABS-KEY ("oral mucositis" OR mucositis OR stomatitis OR "oral complication*" OR "mouth sore*")

==109

Time restriction:
==107
