# Supplementary material for: Nutritional and Supplemental Interventions for Prevention and Treatment of Oral Mucositis in Pediatric Oncology
Source: Nutrients. 2025 Nov 11;17(22):3521. doi: 10.3390/nu17223521 (PMC12655015; doi:10.3390/nu17223521)

Systematic Review

Nutrition and Supplements for Prevention and Treatment of Oral Mucositis in Paediatric Oncology

Supplemental Table S1. Quality Assessment of Included Studies. Y = yes; N = no; CD = cannot determine; NA = not applicable; NR = not reported; RCT=Randomised Controlled Trial.

Author	Study Design	1	2	3	4	5	6	7	8	9	10	11	12	13	14	Quality
Oberbaum 2001 [40]	RCT	Y	CD	Y	Y	Y	Y	Y	Y	Y	Y	Y	NR	CD	Y	Fair
Aquino 2005 [41]	RCT	Y	Y	Y	Y	Y	Y	Y	Y	Y	Y	Y	Y	Y	CD	Good
El-Housseiny 2007 [42]	RCT	Y	Y	Y	Y	Y	Y	N	Y	N	N	Y	N	Y	Y	Fair
Sung 2007 [43]	RCT	Y	Y	NR	Y	N	Y	Y	Y	Y	N	Y	CD	NR	Y	Fair
Ward 2007 [44]	RCT	Y	CD	CD	N	CD	Y	N	Y	Y	Y	Y	NR	Y	N	Fair
Uderzo 2011 [36]	RCT	Y	Y	Y	Y	Y	Y	Y	Y	Y	Y	Y	Y	Y	Y	Good
Abdulrham 2012 [45]	RCT	Y	N	N	N	N	Y	Y	Y	Y	Y	Y	N	Y	Y	Good
Khurana 2012 [46]	RCT	Y	Y	CD	N	Y	Y	Y	Y	Y	Y	Y	N	Y	N	Fair
Sencer 2012 [47]	RCT	Y	Y	Y	Y	N	Y	Y	Y	N	Y	Y	Y	Y	Y	Good
Tomažević 2013 [48]	RCT	Y	CD	Y	Y	Y	Y	N	N	N	Y	Y	N	Y	N	Fair
Raphael 2014 [49]	RCT	Y	Y	Y	Y	Y	Y	Y	Y	Y	Y	Y	N	Y	N	Good
Treister 2016 [37]	RCT	Y	Y	Y	Y	Y	Y	Y	Y	Y	N	Y	Y	Y	Y	Good
Al Jaouni 2017 [50]	RCT	Y	CD	N	N	N	Y	Y	Y	Y	Y	Y	N	Y	Y	Fair
Pourdeghat-kar 2017 [51]	RCT	Y	CD	CD	Y	Y	Y	Y	Y	Y	Y	Y	N	Y	Y	Fair
Alkhouli 2019 [52]	RCT	Y	Y	Y	Y	Y	Y	Y	Y	Y	Y	Y	Y	Y	Y	Good
Rathe 2019 [53]	RCT	Y	Y	Y	Y	Y	Y	Y	Y	N	Y	Y	Y	Y	Y	Good
Widjaja 2020 [54]	RCT	Y	CD	Y	Y	Y	Y	Y	Y	Y	Y	Y	N	Y	Y	Good
Alkhouli 2021 [55]	RCT	Y	Y	Y	Y	Y	Y	Y	Y	Y	Y	Y	Y	Y	Y	Good
Alkhouli 2021 [56]	RCT	Y	Y	Y	Y	Y	Y	Y	Y	Y	Y	Y	Y	Y	N	Good
Badr 2023 [57]	RCT	Y	Y	Y	N	Y	Y	Y	Y	Y	Y	Y	Y	Y	N	Good

Shah 2023 [58]	RCT	Y	Y	Y	Y	Y	Y	Y	Y	Y	Y	Y	Y	Y	Y	Good

Supplemental Figure S1. Risk of bias assessment: Traffic-light plot.

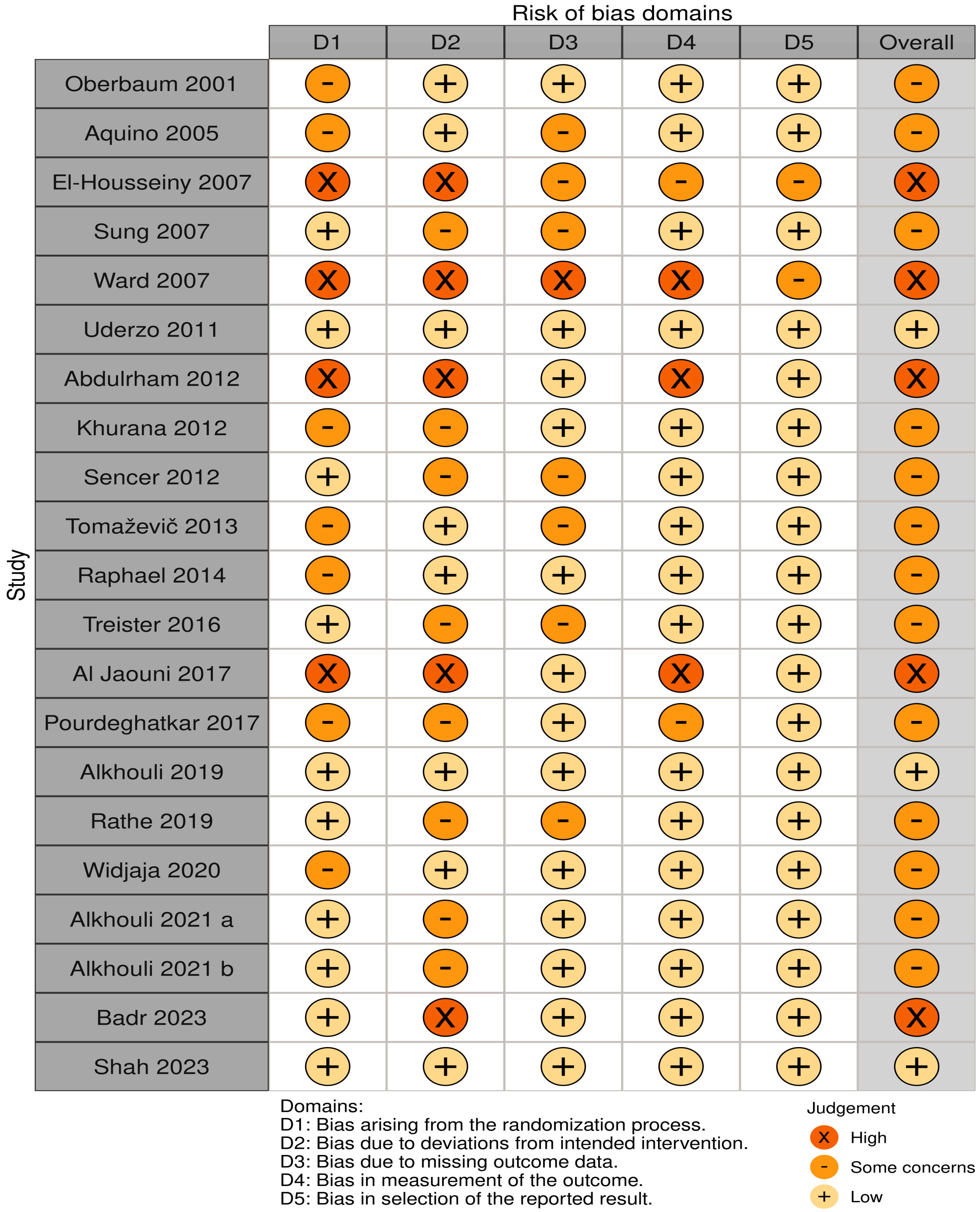

Supplemental Figure S2. Risk of bias assessment: Summary plot.

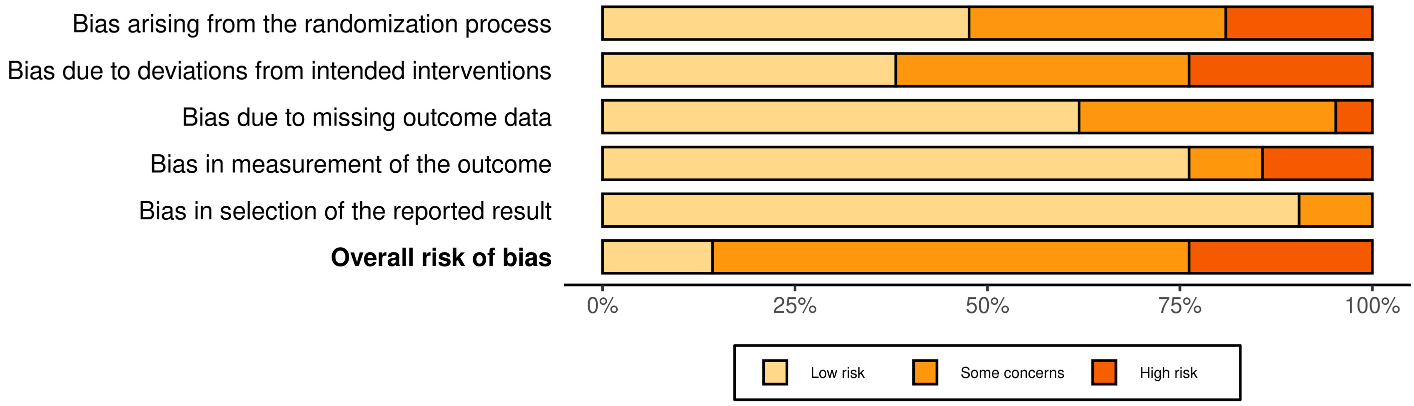

Supplement: Supplementary file 1 [file nutrients-17-03521-s001.zip › nutrients-3923468-supplementary.pdf]
